# Ultrasonographic Considerations for Safe and Efficient Botulinum Neurotoxin Injection in Masseteric Hypertrophy

**DOI:** 10.3390/toxins13010028

**Published:** 2021-01-04

**Authors:** Hyung-Jin Lee, Su-Jin Jung, Seong-Taek Kim, Hee-Jin Kim

**Affiliations:** 1 Division in Anatomy and Developmental Biology, Department of Oral Biology, Human Identification Research Institute, BK21 PLUS Project, Yonsei University College of Dentistry, Seoul 03722, Korea; leehj221@yuhs.ac (H.-J.L.); nannaya221@hanmail.net (S.-J.J.); 2 Department of Orofacial Pain & Oral Medicine, Yonsei University College of Dentistry, Seoul 03722, Korea; k8756050@yuhs.ac; 3 Department of Materials Science & Engineering, Yonsei University College of Engineering, Seoul 03722, Korea

**Keywords:** ultrasonography, botulinum neurotoxin injection, masseter muscle, masseteric hypertrophy, clinical guideline

## Abstract

There are still concerns about masseteric bulging due to a lack of knowledge about the internal architecture of the masseter muscle. Further investigations are therefore required of the most-effective botulinum neurotoxin (BoNT) injection points and strategies for managing masseteric bulging. The purpose of this study was to identify safer and more effective botulinum neurotoxin injection points and strategies by using ultrasonography to determine the structural patterns of the deep inferior tendon. We also measured the precise depths and locations of the deep inferior tendon of the masseter muscle. Thirty-two healthy volunteers participated in this study, and ultrasonography was used to scan the masseter muscle both longitudinally and transversely. Three structural patterns of the deep inferior tendon were identified: in type A, the deep inferior tendon covered the anterior two-thirds of the masseter muscle (21.8%); in type B, the deep inferior tendon covered the posterior two-thirds of the masseter muscle (9.4%); and in type C, the deep inferior tendon covered most of the inferior part of the masseter muscle (68.8%). Depending on the ultrasonography scanning site, the depth from the skin surface to the mandible in the masseteric region ranged from 15 to 25 mm. The deep inferior tendon was typically located 2 to 5 mm deep from the mandible. Ultrasonography can be used to observe the internal structure of the masseter muscle including the deep inferior tendon in individual patients. This will help to reduce the side effects of masseteric bulging when applying retrograde or dual-plane injection methods depending on the structural pattern of the deep inferior tendon.

## 1. Introduction

There is a worldwide tendency of smooth facial lines and a slim jawline being desirable in females, which has resulted in botulinum neurotoxin (BoNT) injections for facial contouring being widely performed ever since this procedure was introduced by Moore in 1994 [1,2,3]. In particular, a hypertrophied masseter muscle is commonly treated in Asians due to the face being more angular than that of Caucasians. The shape of the Caucasian face is narrow compared to that of Asians. Accordingly, a squared jaw is considered more of a major aesthetic issue among Asians than among Caucasians. Angled jawlines are not considered attractive among Asians, especially in young women [4]. For this reason, many previous studies have conducted research to demonstrate the effectiveness of botulinum toxin injections into the masseter muscle in young Asians aged between 20 and 30 [5,6].

The masseter muscle is situated on the lateral side of the face and works strongly with the temporalis muscle during mastication. The lower one-third of the masseter muscle is known to be its thickest part [4]. The conventional BoNT injection technique for the masseter muscle involves performing a deep injection after touching the bone of the mandible, primarily into the lower part [5,7,8]. While BoNT treatment is valuable for alleviating facial asymmetry and nasal flare [9] or facial rhytids of the upper, middle and lower regions of the face [10,11,12,13], several iatrogenic side effects are frequently reported. Avoiding these side effects requires that practitioners have a thorough understanding of the anatomical locations and morphology of the masseter muscle, the relationship between the masseter and risorius muscle, and related vascular structures [14,15,16,17,18]. Masseteric bulging is another side effect, which is observed in 0.49~18.8% of cases and can often occur 2–4 weeks after a BoNT injection [18,19,20]. If the paradoxical masseteric bulging does not recuperate within two weeks, a superficial BoNT injection into the masseter muscle is usually performed [18]. The presence of an abnormal facial expression is another side effect, which is observed in 15~27.3% of cases and which is due to the overspreading of the toxin to the risorius muscle, which is next to the anterior part of the masseter muscle [18,21].

Masseteric bulging can be managed with an additional injection of BoNT, and it usually disappears within 12 weeks. However, frequent BoNT injections result in the production of antibodies that reduce the treatment’s efficacy [22,23,24]. Therefore, various clinical studies have been performed with the aim of identifying BoNT injection points that would avoid masseteric bulging and increase the durability of the treatment effects [20]. These studies of BoNT injections into the masseter muscle have focused on volume changes of the masseter muscle, as evaluated by ultrasonography (US), three-dimensional laser scanning, computed tomography and magnetic resonance imaging (MRI). All of these studies found that injecting BoNT into the masseter muscle was effective for aesthetic purposes. However, there are remaining concerns about masseteric bulging and other side effects due to a lack of knowledge about the internal architecture of the masseter muscle. Further investigations of the most effective BoNT injection points and strategies for managing masseteric bulging are therefore required.

The deep inferior tendon (DIT) of the masseter muscle is located within its superficial part. The previous cadaveric and ultrasonographic study revealed that there were no significant differences between both the male and female DIT morphologies and the right and left sides. However, the most common pattern was different between Korean and Thai cadaveric specimens.

The DIT blocks the BoNT from spreading evenly throughout the superficial masseter muscle, and this tendon can be easily observed in US imaging. Masseteric bulging can be prevented by performing a US-guided injection to accurately locate the depth of the DIT of the masseter muscle.

US can also be used to visualize internal anatomical structures in real time. Although US is less commonly used in the facial area, it is often used for observing masticatory muscles when diagnosing bruxism and temporomandibular joint diseases. Numerous recent clinical and cadaveric studies have investigated the anatomical morphology of the temporomandibular joint and masseter muscle [19,20,25,26].

The purpose of the present study was to identify safer and more effective BoNT injection points and strategies by using US to determine the structural patterns of the DIT, and compare the findings with those from a previous cadaveric study. We also measured the precise depths and locations of the DIT of the masseter muscle.

## 2. Results

### 2.1. Types of DIT in Healthy Young Subjects

**Figure 1:**
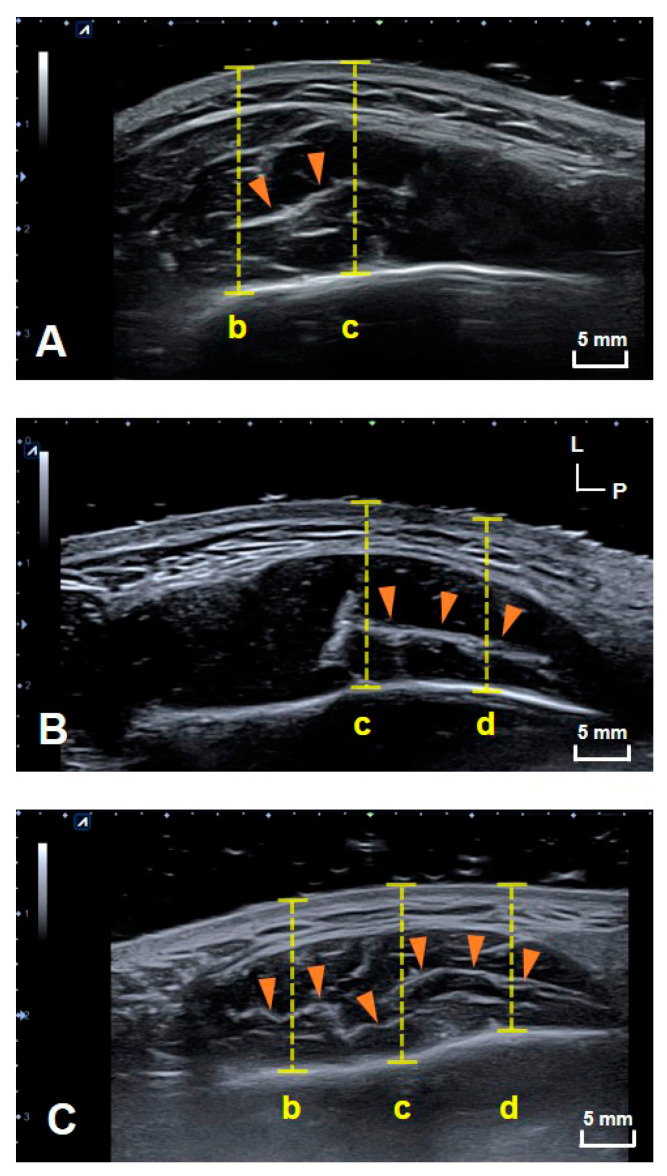
Transverse US images showing the three patterns of the DIT. US images of (**A**) types A, (**B**) B and (**C**) C. (b) The line halfway between the anterior border of the masseter muscle and line c, (c) the line halfway between the anterior and posterior borders of the masseter muscle, and (d) the line halfway between line c and posterior border of the masseter muscle. The yellow dashed lines indicate the depth from the skin surface to the mandible. L, lateral; P, posterior; orange arrowheads, DIT.

US images of the DIT of the masseter muscle were obtained in all subjects on both sides. The structural patterns of the DIT were classified into three different types. Type A covered reference lines b and c (anterior two-thirds of the masseter muscle); type B covered reference lines c and d (posterior two-thirds of the masseter muscle); and type C covered reference lines b, c and d (most of the inferior part of the masseter muscle). Figure 1 shows example images of DITs with structural patterns of types A (21.8%, *n* = 7), B (9.4%, *n* = 3) and C (68.8%, *n* = 22). The DIT could be detected more easily on longitudinal images than transverse images. The classification into the three tendon types did not differ significantly with sex, age, or left and right sides.

### 2.2. Depth at Each Reference Line

**Figure 2:**
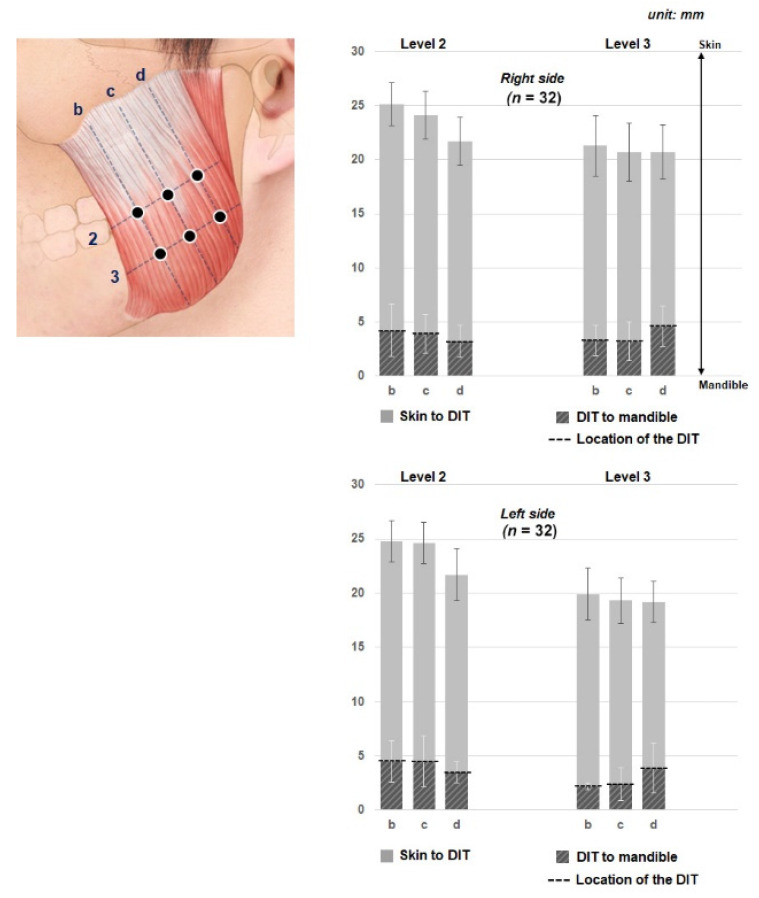
Depths from the skin surface to the DIT and from the DIT to the mandible at each reference line. (b) The line halfway between the anterior border of the masseter muscle and line c, (c) the line halfway between the anterior and posterior borders of the masseter muscle, and (d) the line halfway between line c and posterior border of the masseter muscle. Transverse reference lines include (2) the line halfway between the inferior margin of the zygomatic arch and the inferior margin of the mandible and (3) the line halfway between lines 2 and the inferior margin of the mandible. The black dashed line indicates the location of the DIT within the masseter muscle. Gray indicates the superior part of the DIT in the graphs, which include the thicknesses of the skin, subcutaneous tissue and superficial belly (SB) of the superficial part of the masseter muscle. The DIT could be estimated as being located about 5 mm above the bone surface within the masseter muscle, that is, closer to the mandible than to the skin.

The depths of the DIT at the various reference lines are presented in Figure 2 and Table 1. The DIT tended to be more deeply located in an inferior direction and more superficially in a superior direction. However, the DIT maintained a constant depth transversely in the anterior-to-posterior direction of the masseter muscle (Figure 2).

**Figure 3:**
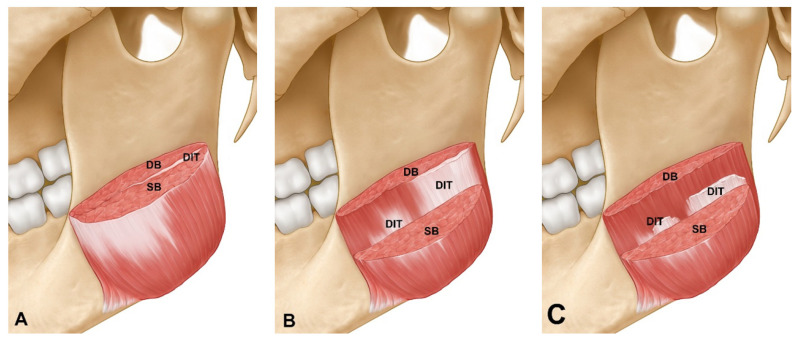
Layer-by-layer depiction of the masseter muscle showing the superficial belly (SB) and deep belly (DB) of the superficial part of the masseter including the deep inferior tendon (DIT). The masseter muscle was cut at the midpoint level between the inferior margins of the zygomatic arch origin and the masseter muscle. (**A**) The DIT divides the superficial part of the masseter muscle into two layers. (**B**) The SB of the superficial part of the masseter muscle is partially removed to reveal the DIT. (**C**) The DB of the superficial part of the masseter muscle is revealed after removing the DIT. The DIT was present about 5 mm above the bone surface within the masseter muscle. Retrograde, dual-plane or US-guided injection methods (depending on the type of DIT) would reduce the side effects of masseteric bulging.

Depending on the US scanning site, the depth from the skin surface to the mandible in the masseteric region ranged from 15 to 20 mm. The DIT was typically located 2 to 4 mm deep from the mandible. The DIT was found to be located about 5 mm above the bone surface within the masseter muscle, that is, closer to the mandible than to the skin (Figure 3).

## 3. Discussion

The present study used US to verify the structural patterns of the DIT of the masseter muscle in healthy young volunteers and compared the findings with those obtained in previous cadaver dissections. Performing US scanning at the same sites as in the cadaveric study has clearly revealed the structural patterns of the DIT. Furthermore, the structural pattern of the DIT was classified into three types: Type A covered references lines b and c (anterior two-thirds of the masseter muscle); type B covered reference lines c and d (posterior two-thirds of the masseter muscle); and type C covered reference lines b, c and d (most of the inferior part of the masseter muscle) (Figure 1).

The previous cadaveric study classified the structural patterns of the DIT into three different types, and similar results were obtained using US [19]. The DIT could be visualized in all subjects, while there were variations in the position of the DIT relative to the reference lines. The DIT was observed at reference lines b and c in 21.8% of the specimens (*n* = 7). While the patterns with reference lines b and c were the least common, in these cases the reference line b should be carefully considered since it is adjacent to the anterior border of the masseter muscle. The anterior border of the masseter coincides with the risorius muscle, which may be located superficially. Consequently, superficial injections into the anterior border area should be performed with the utmost care in order to prevent side effects of changes in the facial muscles due to damage to the risorius. The patterns in which reference lines c and d were covered constituted 9.4% of the specimens (*n* = 3). Reference line d was adjacent to the parotid gland, and any spread of the BoNT to this gland can cause xerostomia or dry mouth. The reported incidence rate for xerostomia is 6.3–13.3%, and the recovery time is typically 3–4 weeks [4,16]. Particular care is therefore recommended when injecting into the posterior part of the masseter muscle, which is the location of reference line d and which is where the parotid gland is located superficial to the masseter muscle. The most common pattern was that where reference lines b, c and d were covered, which was where the superficial part of the masseter muscle divided into the superficial and deep muscle bellies. This pattern was observed in 68.8% of the specimens (*n* =22) and was also the most common pattern that was found in the previous cadaveric study of Korean specimens [20]. Since the DIT divides the superficial part of the masseter muscle into two layers, dual-plane and retrograde injections would be advantageous for preventing masseter bulging.

A consideration of both injection points and depths is crucial to obtaining positive outcomes of masseteric BoNT treatments. Several previous studies have evaluated the durability and formation of the antibodies in the muscle, but they did not reveal the detailed depths of the structures of the masseter muscle, such as of the masseter muscle itself and the DIT. In the present study, the DIT was found at reference lines 2 and 3 of the masseter muscle. At reference line 2, the DIT was generally positioned at a constant depth in the anterior-to-posterior direction. The observations for reference line 3 were similar to those for reference line 2, with the DIT being close to the mandible. Therefore, the depth of the DIT could be estimated as being within 25% of the deeper area of the entire masseter muscle at reference lines 2 and 3. The entire pattern of the DIT could be readily observed in transverse US images as a hyperechoic structure, while longitudinal US images were better for observing the entire presence of the DIT with the masseter muscle.

Various studies have investigated how the intramuscular nerve distribution of the masseter muscle [27], the course of the marginal mandibular branch of the facial nerve [28], and the location, morphology and proportion of the DIT affect the efficacy of injecting BoNT into the masseter muscle [19]. Cioffi et al. reported that the masseter muscle consisted of several aponeuroses that attached to the muscle as a lamellar-like structure, based on observations made using MRI [29]. The present study found that the DIT was present only as a single tendon rather than multiple tendinous structures, as in most cases. However, in some cases the DIT did have more than two tendinous structures. The discrepancy with the description obtained from the previous cadaveric study of Lee et al. [20] is attributable to the use of different research materials and methods. The previous study dissected cadavers and showed only the two-dimensional plane structure of the DIT of the masseter muscle. In contrast, three-dimensional observations are possible using MRI and US. Although in most of the present cases the DIT was observed as a single tendon entering the masseter muscle, the use of US-guided BoNT injections would be helpful for preventing masseteric bulging by confirming the DIT pattern in patients who have multiple DITs.

Previous studies revealed that the internal architecture of the masseter muscle, including the muscle thickness and tendinous structure, varied in the dentate and edentulous group. However, there was no statistical difference between the internal structure of the masseter muscle and age [30]. In addition, our previous studies demonstrated a very similar internal architecture of the masseter muscle between the elderly cadavers and the younger subjects via US [20]. Therefore, the internal architecture of the muscle could be observed as being the same structure within elderly and young age groups. The use of the US is recommended for building patient-specific treatment strategies and/or preventing iatrogenic side effects such as paradoxical masseteric bulging.

Although the present study demonstrated that the internal architecture of the masseter muscle (i.e., DIT, which is helpful when treating BoNT injections in young adults aged approximately 20 to 30), further studies with a larger sample size of various age groups (including elderly ones) are required to build patient-customized treatment strategies for each age group.

## 4. Conclusions

The DIT was observed at a similar depth in the anterior-to-posterior direction, and from the superior to inferior directions it gradually attached to the bone and to the inferior border of the mandible. The DIT was present within 25% of the entire masseter muscle or 5-mm-deep relative to it. In addition, in some cases more than one DIT was observed. Based on this complexity of the structural patterns of the DIT, we believe that US is useful for observing the internal structure of the masseter muscle, including the DIT, in individual patients in real time and will thereby help reduce the side effects of masseteric bulging when applying retrograde or dual-plane injection methods depending on the type of DIT that is encountered.

## 5. Materials and Methods

All of the experimental procedures in this study were performed in accordance with the Declaration of Helsinki of the World Medical Association (version of October 2013). The research was approved by the Institutional Review Board of the Yonsei University Dental Hospital (Approval No. 2-2019-0026, granted on 30 July 2019). A real-time two-dimensional B-mode US system (ECUBE 15, ALPINION Medical Systems, Seoul, Korea) with a 60-mm-wide linear-array transducer (3.0–15.0 MHz; L8-17X, ALPINION Medical Systems) was used to obtain US images of the masseter muscle in healthy young subjects.

While numerous studies have been conducted to elucidate the efficacy of botulinum toxin injection into the masseter muscle, there have been no studies that systematically investigated the age of patients who received botulinum toxin treatment for aesthetic purposes. However, based on previous research that was conducted on a similar topic to this study, most patients were in their 20s [5,6,18]. Therefore, the young age group was selected for the present study.

### US Scanning of Healthy Young Subjects

**Figure 4:**
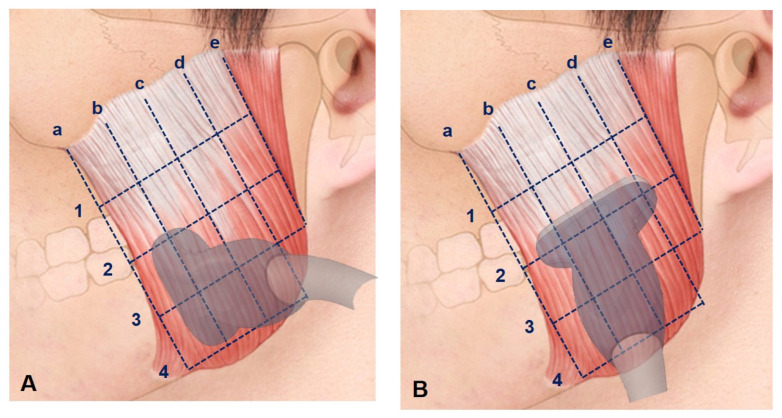
Ultrasonography (US) scanning sites. (**A**) Longitudinal section scanning was performed along reference lines b, c and d of the masseter muscle, while (**B**) transverse section scanning was performed along reference lines 2 and 3. There were five longitudinal reference lines: (a) the anterior border of the masseter muscle, (b) the line halfway between lines a and c, (c) the line halfway between lines a and e, (d) the line halfway between lines c and e, and (e) the posterior border of the masseter muscle. There were four transverse reference lines: (1) the line halfway between the inferior margin of the zygomatic arch and line 2, (2) the line halfway between the inferior margin of the zygomatic arch and line 4, (3) the line halfway between lines 2 and 4, and (4) the inferior margin of the mandible.

Signed written informed consents were obtained from 32 healthy young subjects [16 males and 16 females aged 25.4 ± 4.1 years (mean ± SD)]. The exclusion criteria were orthodontic treatment, temporomandibular joint disorder, plastic surgery or receiving a BoNT injection within the previous six months. The subjects were asked to clench their jaws to ensure that the precise anterior and posterior borders of the masseter muscle could be identified prior to performing US scanning. Longitudinal and transverse reference lines were defined for the US imaging of the masseter muscle (Figure 4). The following five longitudinal reference lines were defined:

- The anterior border of the masseter muscle.
- The line halfway between lines a and c.
- The line halfway between lines a and e.
- The line halfway between lines c and e.
- The posterior border of the masseter muscle.

The following four transverse reference lines were defined:

1. The line halfway between the inferior margin of the zygomatic arch and line 2.
2. The line halfway between the inferior margin of the zygomatic arch and line 4.
3. The line halfway between lines 2 and 4.
4. The inferior margin of the mandible.

The US sampling frequency was set to 15.0 MHz, which is an ideal frequency for observing depths between 2.5 and 4 cm depending on the presence of skin, fat and muscle tissues. A water-soluble gel was applied to the skin to optimize the acquisition of images and prevent artifacts. The US transducer was positioned perpendicularly to the skin surface over the area of the masseter muscle. The imaging was performed on both sides of the face. The DIT of the masseter muscle and its location were confirmed by cross-checking between the transverse and longitudinal US images.

**Figure 5:**
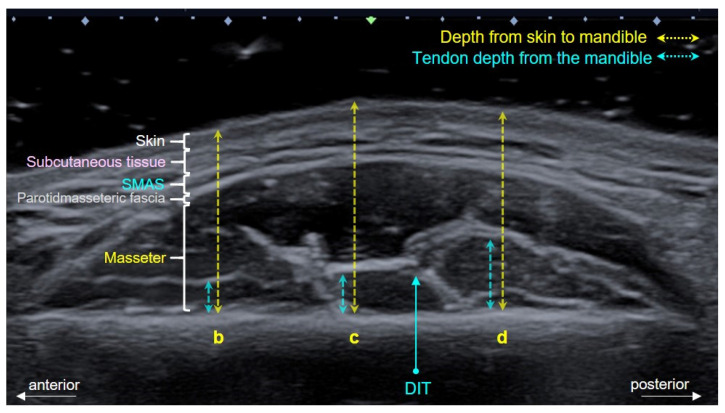
Transverse US image showing the masseteric area, including the deep inferior tendon (DIT) within the muscle. The depths from the skin surface to the mandible (yellow dashed line) and from the mandible to the DIT (blue dashed line) at reference lines b, c and d were measured on the transverse US image. SMAS, superficial musculo-aponeurotic system.

The morphology of the DIT was classified based on the previous cadaveric study that divided the tendon into three types. Types A, B and C covered references lines b and c (anterior two-thirds of the masseter muscle), reference lines c and d (posterior two-thirds of the masseter muscle), and reference lines b, c and d (most of the inferior part of the masseter muscle), respectively. The depths from the skin to the mandible and from the mandible to the DIT at each reference line were measured using Image J software (National Institutes of Health, Bethesda, MD, USA) (Figure 5).

## Figures and Tables

**Table 1:** Depths (in millimeters) from the skin surface to the mandible and from the DIT (deep inferior tendon) to the mandible on the left and right sides.

	2 (*n* = 32)				3 ( *n* = 32)			
	Skin to DIT		DIT to Mandible		Skin to DIT		DIT to Mandible	
	Right	Left	Right	Left	Right	Left	Right	Left
b	20.9 ± 2.0	20.3 ± 1.9	4.2 ± 2.4	4.5 ± 1.9	18.0 ± 2.8	17.7 ± 2.4	3.3 ± 1.4	2.2 ± 0.3
c	20.2 ± 2.2	20.1 ± 1.9	3.9 ± 1.8	4.5 ± 2.4	17.5 ± 2.7	16.9 ± 2.1	3.2 ± 1.8	2.4 ± 1.5
d	18.5 ± 2.2	18.2 ± 2.4	3.2 ± 1.5	3.5 ± 1.0	16.1 ± 2.5	15.3 ± 1.9	4.6 ± 1.9	3.9 ± 2.3

2, the line halfway between the inferior margins of the zygomatic arch and of the masseter muscle; 3, the line halfway between line 2 and the inferior margin of the mandible; b, the line halfway between the anterior border of the masseter muscle and line c; c, the line halfway between the anterior and posterior borders of the masseter muscle; d, the line halfway between line c and the posterior border of the masseter muscle.
